# Institutional Questions

**Published:** 1900-02-10

**Authors:** 


### INSTITUTIONAL QUESTIONS.

### The Mary Wardell Convalescent Home.

"F. P." writes: Please give me some particulars of the Mary Wardell Convalescent Home. Is it for paying patients only?

*** The Mary Wardell Convalescent Home for Scarlet Fever is at Stanmore, Middlesex. It has 40 beds, and is for women and girls, and boys under 12. Only paying patients are admitted, payment being from 15s. a week for women and 10s. for children to £3 3s. for private patients. Write for all particulars to the matron, Miss Cahusac.

### Bed Tables.

"Secretary" asks: The committee of my hospital have sanctioned the purchase of a certain number of bed tables for our wards. I shall be glad if you can tell me whether there is any specially new design lately adopted more to be recommended than any other?

*** The best design for bed tables we have recently seen is that adopted at the London Hospital and other metropolitan institutions. The table stands on the floor and is high enough and wide enough to go easily over the bed, supported on both sides, and the legs mounted on castors, so that the whole things runs easily. We should suggest your paying a visit to the London or Westminster Hospital, where some of this kind have recently been provided, and examining these tables for yourself. They have naturally many advantages over the rather unsteady little articles intended to stand on the bed, even if they take up somewhat more room.

### Architects of Cottage Hospitals.

"A Correspondent" writes: It is proposed to enlarge or rebuild a small hospital in which I am interested which at present contains nine beds. Can you give me the name of two architects who have done hospital work on a similar scale to that required, *i.e.*, to contain fifteen or sixteen beds?

*** We think your best course would be to obtain a copy of Burdett's "Cottage Hospitals" (London: The Scientific Press, 28 and 29, Southampton Street, price 5s.), where you will find the plans of the most recent and approved hospitals of the type you indicate, and also the name of the architect in each case.

